# Influence of different immersion media on the color stability of resin-based composites

**DOI:** 10.4317/jced.64382

**Published:** 2026-09-15

**Authors:** Ahmad Yousef Alkhodair, Rodrigo R Maia, Gisele Neiva, Dayane Oliveira, Mateus Rocha

**Affiliations:** 1 Department of Cariology, Restorative Sciences and Endodontics, University of Michigan - School of Dentistry, Ann Arbor, Michigan, USA; 2 Restorative and Prosthetic Dental Science Department, College of Dentistry, King Saud Bin Abdulaziz University for Health Sciences, Riyadh, 14611, Saudi Arabia; 3 Department of Restorative Dental Sciences, University of Florida - College of Dentistry, Gainesville, Florida, USA

**Keywords:** Resin-based composites, color stability, CIEDE2000, universal-shade composite, sports drinks, energy drinks.

## Abstract

**Background:**

Color instability continues to be a major reason for replacing composite restorations. Sports and energy drinks contain low pH, artificial colorants, and high sugar levels; however, their impact on universal-shade resin-based composites (RBCs) is still not well understood.

**Materials and Methods:**

Seventy-two disc-shaped specimens (10 mm diameter × 1 mm thickness; n=12 per group) of two universal-shade RBCs, Omnichroma (Tokuyama Dental) and Transcend Universal (Ultradent Products), were fabricated and light-cured at a verified irradiance of 873 mW/cm^2^ for 20 seconds. Pre-immersion measurements served as individual reference baselines per ISO 4049:2019. Specimens were immersed in C4 Energy Drink (Bombsicle), Gatorade (Lemon Lime), or water for 1, 3, and 7 days. Color was measured using a Konica Minolta CM-700d spectrophotometer with a custom silicone putty jig and 3 mm aperture of spectrophotometer. Data was recorded with SpectraMagic NX2 software. Overall color change was calculated as ΔE00 (CIEDE2000), and data were analyzed by three-way ANOVA with Tukey HSD post-hoc comparisons (α=.05).

**Results:**

Composite type, immersion medium, and time point each significantly affected ΔE00 (all p<.001). Transcend showed greater overall color change than Omnichroma (1.942 ± 0.056 vs. 1.603 ± 0.056; p<.001). C4 and Gatorade produced comparable ΔE00 values (both significantly greater than water) but opposite chromatic shifts along the b* axis: Gatorade toward yellow (Δb* ≈2.4) and C4 toward blue (Δb* ≈-2.2). Transcend in both beverages exceeded the ΔE00 ≈3.6 level (twice the 50:50% acceptability threshold) by Day 7; Omnichroma remained within the moderate-match range.

**Conclusions:**

Both composites showed measurable, progressive discoloration. Omnichroma outperformed Transcend in color stability, a difference possibly related to its more hydrophobic UDMA-based matrix and structural-color filler mechanism. Beverages producing similar ΔE00 magnitudes can cause distinct chromatic shifts, supporting evidence-based material selection for patients exposed to acidic or chromogenic beverages.

**Introduction** 

The repair and replacement of damaged tooth structure has driven major advances in restorative materials [1]. Dental caries remains one of the most common chronic diseases worldwide, creating a substantial public health and economic burden [2]. The shift from amalgam toward tooth-colored polymer-based materials was accelerated by Bowen’s synthesis of Bis-GMA in the 1960s [3] and has intensified following the Minamata Convention phase-down of dental amalgam [4,5]. 

RBCs consist of an organic resin matrix and an inorganic filler phase linked through silane coupling agents [6]. The organic matrix typically contains Bis-GMA combined with triethylene glycol dimethacrylate (TEGDMA) as a viscosity-reducing diluent, together with a camphorquinone photoinitiator [7]. The inorganic filler phase (silica, quartz, zirconia, or ceramic particles) reinforces the material, improves wear resistance, reduces polymerization shrinkage, and governs optical properties [6]. Based on filler particle size and distribution, composites are classified as macrofill, microfill, hybrid/microhybrid, and nanofill materials, each providing a different balance of strength, polishability, and esthetics [8,9]. 

Despite their clinical advantages, color stability is among the primary esthetic concerns for RBCs and is key to long-term patient satisfaction [10]. Color is quantified using the CIELAB color space (L*, a*, b*) [10], and the CIEDE2000 formula (ΔE_00_) provides a perceptually uniform metric for clinical color-change assessment [11,12]. Small differences in CIELAB values can be detected visually, and values exceeding accepted thresholds indicate clinically perceptible color change [10,13]. 

A new generation of universal-shade RBCs has emerged to simplify shade matching. Omnichroma (Tokuyama Dental, Japan) generates structural color through uniformly sized 260-nm supra-nanospherical silica zirconia fillers that selectively scatter wavelengths to match the surrounding tooth structure. Transcend Universal (Ultradent Products Inc., USA) achieves universal shade matching through its Resin Particle Match™ technology, which optimizes filler-matrix refractive-index matching. Both represent structurally distinct approaches to shade universality, yet their comparative color stability under acidic dietary challenge has not been established. 

Sports and energy drinks are of particular clinical relevance: They are among the most frequently consumed beverages among younger adults, the core demographic for esthetic composite restorations, and combine low pH, synthetic colorants, and high sugar content in a profile mechanistically distinct from the tannin-based staining associated with coffee and tea [14]. Despite their wide consumption among this demographic, sports and energy drinks remain under-studied as staining challenges for universal-shade composites relative to coffee and tea, providing the rationale for the present comparison. The present work therefore addresses two gaps: It directly compares two structurally distinct universal-shade composites under identical conditions, and it characterizes their behavior under acidic sports and energy drinks rather than the coffee and tea that dominate prior staining studies. 

This study aimed to evaluate and compare the color stability of Omnichroma and Transcend after immersion in C4 Energy Drink (Bombsicle flavor) and Gatorade (Lemon Lime flavor), with water as the standard ISO 4049:2019 storage medium, at four time points. The null hypotheses were: (H01) no significant difference in ΔE_00_ between composite materials; (H02) no significant difference in ΔE_00_ across time points. 

## Materials and Methods 

*Study design*

A pilot study was conducted prior to data collection to estimate the appropriate sample size. A priori power analysis using G*Power 3.1 (ANOVA: Fixed effects, main effects and interactions; effect size *f*=0.40; α=0.05; 6 groups) confirmed that a sample of 72 specimens provided statistical power above 80% (actual power = 0.804). The 72 disc-shaped specimens were allocated to six experimental groups (2 composites × 3 immersion media; n=12 per cell), assessed at four color measurement points (baseline, Day 1, Day 3, Day 7). The materials and immersion media are summarized in Table 1. 

*Ethics statement*

This study was an *in vitro* investigation of dental restorative materials. No human participants, human tissue, or animal subjects were involved; therefore, Institutional Review Board approval was not required. 

*Specimen preparation*

Disc-shaped specimens (10.0 mm diameter × 1.0 mm thickness) were fabricated using a custom aluminum mold. A thickness of 1.0 mm was selected based on established optical frameworks: Kubelka-Munk scattering theory predicts sufficient internal scattering at this thickness to mask the underlying substrate, and both Translucency Parameter (TP) and Contrast Ratio (CR) values for RBCs at 1 mm indicate that this thickness minimizes, but may not entirely eliminate, the influence of the underlying background, consistent with Kim *et al*. [15] and Vichi *et al*. [16]. All color measurements were performed against a standardized white background. 

Composite material was placed into each mold well, and a glass microscope slide was pressed over the mold until the composite uniformly filled the well. Light curing was performed using the Ultradent Valo (corded) polywave light-curing unit (wavelength range 385-515 nm; peak emissions 395-415 nm and 440-480 nm). A custom jig maintained standardized angulation and distance, and irradiance was verified at 873 mW/cm² using a calibrated radiometer (Gigahertz-Optik MSC-15W) before and after each preparation group [17]. All specimens were light-cured for 20 seconds. Following polymerization, specimens were stored in distilled water at 37°C for 24 hours to allow complete post-cure and material stabilization, then gently dried with tissue paper before baseline color measurement. No additional polishing was applied; the glass Mylar interface produced a consistent smooth surface across all groups. 

*Baseline color measurement and ISO controls*

All 72 specimens were measured individually with a calibrated Konica Minolta CM-700d spectrophotometer before immersion. To ensure standardized, reproducible, and environment-independent measurements, a custom silicone putty jig was fabricated to hold each specimen in a fixed position and to precisely mount the spectrophotometer over the specimen. The 3 mm small-aperture measurement port was used for all readings to restrict the measurement field to the specimen surface and eliminate influence from the surrounding environment. All color measurements were performed under identical, standardized light conditions, and data were recorded and analyzed using SpectraMagic NX2 software (Konica Minolta). Measurements were obtained in the specular-component-included (SCI) mode using the instrument’s d/8° geometry (diffuse illumination, 8° viewing) with the CIE D65 illuminant and 2° standard observer. The SCI mode was selected because, by including the specular component, it captures the intrinsic color of the material independent of surface gloss and texture, rather than the gloss-influenced color perceived by the human eye that the specular-component-excluded (SCE) mode represents; this isolates color change attributable to the composite itself rather than to surface-appearance effects. CIELAB coordinates (L*, a*, b*) were recorded for each specimen. The spectrophotometer was calibrated against its white calibration plate and zero-calibration box before each measurement session. 

In accordance with ISO 4049:2019, the pre-immersion CIELAB values of each specimen served as its individual reference baseline [18]. Distilled water was used as the standard storage medium to quantify color change attributable solely to water sorption and hydrolytic aging, against which the additional chromogenic effects of the sports and energy drinks were compared. 

*Experimental groups and storage conditions*

Following baseline measurement, specimens were divided into six groups (Table 1; Figure 1): OW (Omnichroma + water), OG (Omnichroma + Gatorade), OC (Omnichroma + C4 Energy), TW (Transcend + water), TG (Transcend + Gatorade), TC (Transcend + C4 Energy). Specimens were stored in 500 mL HDPE UV-excluding containers, individually labeled, and maintained at room temperature throughout the immersion period. Immersion media were replaced with freshly prepared solutions at each measurement time point (Day 1, Day 3, and Day 7) to maintain consistent chromogen concentration and pH. 

*Color-change assessment*

Color measurements were recorded at baseline and after 1, 3, and 7 days of continuous immersion. These time points represent an accelerated-aging model. Guler *et al*. reported that 24 hours of continuous immersion simulated approximately 1 month of beverage consumption, based on an average drinking time of 15 minutes per cup and 3.2 cups per day in coffee drinkers [19]. Adapting this framework to a single-serving sports or energy drink exposure of approximately 20 minutes per day, 1 day of continuous immersion approximates approximately 2.4 months of clinical beverage exposure, 3 days approximately 7.2 months, and 7 days approximately 16.8 months. These conversions should be interpreted cautiously, as continuous immersion does not reproduce the intermittent exposure, salivary clearance, and dilution of the oral environment and therefore likely overestimates real-world clinical exposure. At each time point, specimens were removed from their containers, rinsed with distilled water, gently dried with tissue paper, repositioned in the silicone putty jig, and remeasured under identical light conditions. The overall color change (ΔE_00_) was calculated using the CIEDE2000 formula as described by Sharma *et al*. [12], which incorporates perceptual weighting functions for lightness, chroma, and hue differences to provide a more uniform color-difference metric than the earlier CIELAB formula. The CIEDE2000 color difference was computed as ΔE_00_ = √[(ΔL′/SL)² + (ΔC′/SC)² + (ΔH′/SH)² + RT(ΔC′/SC)(ΔH′/SH)], where ΔL′, ΔC′, and ΔH′ are the lightness, chroma, and hue differences; SL, SC, and SH are the weighting functions; and RT is the rotation term [12]. 

*Statistical analysis*

Raw color data were entered into Microsoft Excel; inferential analyses were performed using IBM SPSS Statistics Version 30.0 (α=.05). A three-way between-subjects ANOVA evaluated the main effects and all two- and three-way interactions of composite material (Omnichroma vs. Transcend), immersion medium (water, Gatorade, C4), and time point (baseline, Day 1, Day 3, Day 7) on ΔE_00_, ΔL*, Δa*, and Δb*. Tukey HSD post-hoc tests were applied for all significant effects. In total, 240 ΔE_00_ observations (120 per composite) were analyzed across the four time points. Effect sizes are reported as partial *η*² (≥.01 small, ≥.06 medium, ≥.14 large). 

**Results** 

*Baseline color values*

Prior to immersion, mean ΔE_00_ values at baseline (T0) were 0.648 ± 0.238 for Omnichroma and 1.143 ± 0.613 for Transcend (n=12 each). These values represent the inherent within-group color variation of each material under measurement conditions, not post-immersion color change. All subsequent ΔE_00_ values reported are calculated relative to each specimen’s individual baseline measurement, ensuring that any pre-existing color variation between materials does not confound the immersion results. 

*Overall color change (ΔE*_00_ *)*

The three-way ANOVA on ΔE_00_ yielded a significant corrected model, *F*(19, 220)=30.638, *p*<.001, *R*²=.726 (Adjusted *R*²=.702). All three main effects were significant: Composite type, *F*(1, 220)=18.048, *p*<.001, partial *η*²=.076 (medium); time point, *F*(2, 220)=67.942, *p*<.001, partial *η*²=.382 (large); immersion medium, *F*(2, 220)=103.013, *p*<.001, partial *η*²=.484 (large). All two-way interactions were significant: Composite × Time (*F*(2, 220)=34.030, *p*<.001, partial *η*²=.236), Composite × Medium (*F*(2, 220)=19.416, *p*<.001, partial *η*²=.150), and Time × Medium (*F*(4, 220)=11.414, *p*<.001, partial *η*²=.172). The three-way Composite × Time × Medium interaction was also significant (*F*(4, 220)=3.679, *p*=.006, partial *η*²=.063). Estimated marginal means and Tukey HSD groupings for all main effects are reported in Table 2 (Figure 2). 

Transcend showed significantly greater color change than Omnichroma (EMM: 1.942 ± 0.056 [95% CI 1.832-2.053] vs. 1.603 ± 0.056 [1.493-1.714]; mean difference 0.339, 95% CI [0.183, 0.496], Bonferroni *p*<.001). 

Time point. Estimated marginal means increased from baseline (T0: 0.896 ± 0.126 [0.648-1.143]) through Day 1 (T1: 1.220 ± 0.072 [1.077-1.363]), Day 3 (T2: 1.995 ± 0.072 [1.853-2.138]), and Day 7 (T3: 2.395 ± 0.072 [2.252-2.538]). T0 and T1 did not differ significantly (Bonferroni *p*=.157); T2 and T3 were each significantly higher than both T0 and T1 (all *p*<.001), and T3 was significantly higher than T2 (*p*<.001), indicating continued accumulation of color change through Day 7. Medium. C4 (2.309 ± 0.072 [2.166-2.451]) and Gatorade (2.281 ± 0.072 [2.138-2.424]) were each significantly greater than water (1.021 ± 0.072 [0.878-1.164]; Bonferroni *p*<.001) and did not differ from each other (*p*=1.000). 

Lightness, red-green, and yellow-blue changes (ΔL*, Δa*, Δb*)

The three-way ANOVA on ΔL* was significant, *F*(19, 220)=10.555, *p*<.001, *R*²=.477. Main effects were significant for composite type (*F*(1, 220)=10.773, *p*=.001, partial *η*²=.047), time (*F*(2, 220)=21.427, *p*<.001, partial *η*²=.163), and medium (*F*(2, 220)=20.160, *p*<.001, partial *η*²=.155). Omnichroma showed net lightening across the study (+0.779 ± 0.118); Transcend was substantially less lightened (+0.225 ± 0.118; Bonferroni *p*=.001). Among time points, ΔL* peaked at Day 3 (T2: +1.221 ± 0.153) then reversed, with Day 7 (T3: -0.178 ± 0.153) not significantly different from baseline (T0 vs T3: *p*=1.000), indicating a return toward pre-immersion lightness. Among media, Gatorade produced the largest lightening (EMM +1.345 ± 0.153); C4 (+0.353 ± 0.153) and water (+0.031 ± 0.153) showed smaller or negligible shifts. 

The three-way ANOVA on Δa* was significant, *F*(19, 220)=44.898, *p*<.001, *R*²=.795. Composite type was not significant (*F*(1, 220)=0.136, *p*=.712, partial *η*²=.001). The Time × Medium interaction was exceptionally large (*F*(4, 220)=101.804, *p*<.001, partial *η*²=.649). C4 produced a shift toward red (EMM +0.238 ± 0.026) while Gatorade shifted toward green (-0.239 ± 0.026; Bonferroni *p*<.001), reflecting their respective dye compositions. 

The three-way ANOVA on Δb* was significant, *F*(19, 220)=63.309, *p*<.001, *R*²=.845. Immersion medium accounted for the dominant share of variance (*F*(2, 220)=411.968, *p*<.001, partial *η*²=.789). C4 produced a strong shift toward blue (EMM -2.205 ± 0.115), Gatorade the largest shift toward yellow (+2.403 ± 0.115), and water a smaller positive shift (+0.745 ± 0.115); all pairwise medium differences were significant (all *p*<.001). Composite type was significant (*F*(1, 220)=20.799, *p*<.001, partial *η*²=.086): Omnichroma showed a net positive Δb* (+0.595 ± 0.089) while Transcend was slightly negative (-0.084 ± 0.089; *p*<.001). For time, only T2 (Day 3: +0.577 ± 0.115) differed significantly from T0 (*p*=.002) and T1 (*p*=.005); T3 (Day 7: +0.337 ± 0.115) did not differ significantly from any other time point (all *p*>.05). Mean values and Tukey groupings for ΔL*, Δa*, and Δb* by composite and medium, collapsed across time points, are presented in Table 3. The time-resolved main-effect values reported above capture the temporal pattern, including the Day 3 peak and Day 7 reversal in ΔL*, that Table 3 does not display. 

*Cell-level ΔE*_00_ *and clinical classification*

Transcend in Gatorade (TG: 3.757 ± 0.505 [95% CI 3.407-4.107] at Day 7) and Transcend in C4 (TC: 3.708 ± 1.041 [3.358-4.058] at Day 7) exceeded the ΔE_00_ ≈3.6 level, the boundary between moderately and clearly unacceptable mismatch in the Paravina *et al*. [10] interpretation model, progressing from Excellent at Day 1 (TG: 0.613 ± 0.292; TC: 1.592 ± 0.338) to Clearly Unacceptable by Day 7. Omnichroma remained in the Moderate range for both beverages at Day 7 (OG: 2.289 ± 0.313 [1.939-2.639]; OC: 2.090 ± 0.821 [1.740-2.440]). All water-immersed groups (OW, TW) remained within the Acceptable range throughout (OW Day 7: 1.239 ± 0.297; TW Day 7: 1.288 ± 0.752). Cell-level values with Paravina clinical classifications are presented in Table 4 (Figure 3). 

**Discussion** 

*Overview*

This *in vitro* study compared the color stability of two universal-shade RBCs after immersion in C4 Energy Drink, Gatorade, and water over 7 days. Three findings stand out: Transcend showed significantly greater overall color change than Omnichroma; C4 and Gatorade produced similar ΔE_00_ magnitudes but opposite chromatic shifts; and discoloration increased progressively through Day 7, with Transcend reaching Clearly Unacceptable by Day 7 in both beverages. 

*Staining mechanisms: Adsorption and diffusion*

Discoloration of RBCs in chromogenic media proceeds through two sequential but overlapping mechanisms: Surface adsorption and bulk diffusion [19,20]. 

Surface adsorption dominates the early phase of immersion. Chromogen molecules bind to polar groups and surface irregularities via van der Waals forces, hydrogen bonding, and electrostatic interactions [14,19]. This process is mainly driven by surface energy and accounts for most of the color changes seen at Day 1. Even on the glass-pressed smooth surfaces used here, dye adsorption still occurred, indicating that chemical affinity, not surface roughness, drives early-phase color change. 

Bulk diffusion becomes the dominant mechanism during sustained immersion. Water molecules penetrate the polymer network and plasticize the cross-linked matrix by disrupting intermolecular hydrogen bonds, increasing segmental mobility and free volume [20]. This expanded free volume creates molecular-scale pathways through which chromogen molecules migrate from the surface into the composite matrix. At the same time, the acidic pH of both beverages (C4 ≈3.0-3.5; Gatorade ≈2.9-3.2) accelerates hydrolysis of ester bonds within the resin matrix and at the silane coupling layer of the resin-filler interface [21], generating microcracks that serve as larger-diameter preferential diffusion channels [20,21]. The steady increase in ΔE_00_ from Day 1 through Day 7, with no plateau, points to diffusion-dominated kinetics sustained by a persistent concentration gradient. At Day 1, the Composite × Medium interaction was not significant (*p*=.118), but it became highly significant at Day 3 and Day 7 (both *p*<.001), indicating that adsorption affected both composites similarly early on while the later divergence reflects material-dependent resistance to bulk diffusion. 

*Why Omnichroma outperformed Transcend*

Omnichroma’s superior color stability may be related to two material-level factors: A more hydrophobic resin matrix and greater filler-mediated optical resilience. 

Starting with resin matrix chemistry, Omnichroma contains a UDMA- and TEGDMA-based matrix, whereas 

Transcend uses dimethacrylate and methacrylate resins. UDMA lacks the pendant hydroxyl groups present in Bis-GMA, making the network more hydrophobic and less prone to water uptake [22]. Fonseca *et al*. demonstrated that UDMA-containing formulations exhibit significantly lower water sorption and solubility than Bis-GMA-based systems [22], which directly limits the matrix plasticization that drives bulk chromogen diffusion. Huang *et al*. confirmed a statistically significant positive correlation between water sorption and ΔE_00_ across 11 commercial composites (*p*<.001) [23]. By resisting water uptake, Omnichroma’s matrix may limit free-volume expansion and thereby reduce the speed and magnitude of chromogen penetration. These findings are consistent with Duzyol *et al*. and El-Rashidy *et al*., who reported lower discoloration for UDMA-rich single-shade composites compared with Bis-GMA-dominant formulations [24,25]. 

Turning to filler morphology, Omnichroma’s uniform 260-nm supra-nanospherical silica-zirconia fillers operate in the Mie scattering regime, selectively scattering wavelengths matching the surrounding tooth structure [26]. This color mechanism originates within the composite bulk from filler geometry and size, not from surface pigmentation. When chromogen molecules accumulate on the surface through adsorption, the filler-driven scattering signal may continue to dominate the spectrophotometric output, potentially masking the chromatic shift from surface-adsorbed dye through an optical compensation effect attributed to the spherical filler geometry. In contrast, Transcend’s Resin Particle Match™ technology relies on minimizing the filler-matrix refractive-index mismatch in the cured state. Acid-mediated hydrolysis of the silane coupling layer may disrupt this refractive-index balance, possibly increasing internal light scattering at the filler-matrix interface and compounding the chromatic shift produced by bulk chromogen diffusion [20,21]. These findings agree with Janson *et al*. [27] and Abdul Kareem *et al*. [28], and extend those observations to acidic sports and energy drinks. More broadly, the disc-specimen, CIEDE2000, beverage-immersion paradigm used here belongs to a well-established body of *in vitro* research on single-shade composites: Omnichroma has been compared with other color-adjustment and conventional composites in cola, coffee, and black tea [24,29], after thermocycling in tea and coffee [28], and against Vittra APS Unique in turmeric, tea, and soft drinks [30]. The present study does not replicate any single previous investigation. It is unique because it focuses on acidic sports and energy drinks, directly compares their effects on both Omnichroma and Transcend Universal, and shows that two beverages can produce nearly identical overall ΔE_00_ values yet shift the color in completely opposite directions, a difference that cannot be detected when ΔE_00_ is the only metric reported. It should be emphasized that the present study measured only net color change (ΔE_00_ and CIELAB coordinates); the structural-color and Mie-scattering masking effects described here are mechanistic explanations derived from the optical literature and manufacturer data rather than phenomena directly characterized in this work, and should be regarded as hypotheses requiring further investigation. 

*Beverage-driven chromatic shifts*

C4 and Gatorade produced statistically equivalent ΔE_00_ values (Bonferroni *p*=1.000), yet their contributions along the CIELAB axes differed markedly. Gatorade produced a strong positive Δb* toward yellow (EMM +2.403 ± 0.115), consistent with Yellow #5 and Yellow #6; C4 produced a strong negative Δb* toward blue 

(EMM -2.205 ± 0.115) consistent with Blue 1, and a mild positive Δa* toward red (+0.238 ± 0.026; Red 40). These opposing shifts matter clinically. Two beverages can produce the same ΔE_00_ magnitude yet cause entirely different visible hue changes, which is relevant to both patient counseling and the timing of shade re-evaluation. This dissociation between the magnitude and the direction of color change may represent one of the most clinically relevant findings of this study, indicating that ΔE_00_ magnitude alone may not fully capture clinically meaningful differences in the appearance of discoloration; the directional CIELAB shift should therefore be considered alongside ΔE_00_ when anticipating esthetic outcomes. 

The ΔL* reversal between Day 3 and Day 7 fits a transition from early surface phase hydrolytic change to later bulk chromogen accumulation. Hydrolytic degradation of the resin matrix and silane interface during early immersion can alter near-surface optical behavior, whereas continued diffusion of beverage colorants into the composite bulk increasingly contributes to light absorption over time [20,21]. Given that the effect size for ΔL* was modest relative to the much larger medium effect on Δb*, this lightness reversal is a secondary phenomenon, not the main driver of overall discoloration. More broadly, several statistically significant differences in individual CIELAB coordinates particularly small ΔL* and Δa* shifts fell below the 0.8 perceptibility threshold and are therefore unlikely to be clinically perceptible; statistical significance under the present design should not be equated with clinical relevance. 

*Clinical implications*

Within the limitations of this *in vitro* study, the findings suggest that frequent exposure to acidic, dye-containing sports and energy drinks may affect the color stability of universal-shade resin-based composites. Under the tested conditions, Omnichroma demonstrated lower color change than Transcend Universal; however, these results should not be directly interpreted as definitive clinical performance. The magnitude of ΔE_00_ and the direction of chromatic shifts represent distinct aspects of color change and should be considered separately when evaluating esthetic outcomes. Further *in vivo* and long-term clinical studies are needed to confirm the clinical relevance of these findings. 

Clinicians should consider patients’ dietary habits, particularly frequent consumption of acidic or chromogenic beverages, when selecting restorative materials and counseling patients regarding the maintenance of esthetic composite restorations. 

*Limitations*

This study was conducted *in vitro* and therefore cannot fully reproduce the complexity of the oral environment. Factors such as salivary pellicle formation, salivary buffering, enzymatic activity, mastication, plaque biofilm, and variations in oral hygiene were not simulated. Thermocycling and toothbrushing simulation were also not performed. In addition, the continuous immersion protocol represents an accelerated aging model and does not fully reflect the intermittent exposure patterns that occur clinically. Only two universal-shade resin-based composites, each tested in its single universal shade, and two beverage formulations were evaluated; the limited number of materials and brands, together with the *in vitro* design, constrains direct extrapolation to clinical performance. Future studies incorporating water sorption, solubility, surface roughness, and surface morphology analyses would strengthen the mechanistic interpretation of the observed color changes. In addition, optical parameters central to the behavior of universal-shade composites including the translucency parameter, contrast ratio, whiteness index, opalescence, and surface gloss were not measured, nor was beverage color objectively characterized; their assessment in future work would further clarify the optical basis of the observed color changes. 

**Conclusions** 

Within the limitations of this *in vitro* study, the following conclusions are drawn: Both composites showed measurable and progressive discoloration throughout the 7-day testing period, with no evidence of a plateau; Omnichroma demonstrated significantly better color stability than Transcend Universal, rejecting H01. This advantage may be attributable to its UDMA-based hydrophobic matrix and the optical resilience of its structural-color filler mechanism; C4 Energy Drink and Gatorade produced similar overall ΔE_00_ magnitudes but caused opposite chromatic shifts (Δb*) reflecting their distinct dye compositions; Transcend exceeded the ΔE_00_ ≈3.6 clinically unacceptable level by Day 7 in both beverages (Gatorade: 3.76 ± 0.50; C4: 3.71 ± 0.33), while all Omnichroma specimens remained in the Moderate range, rejecting H02; and these findings support resin chemistry, filler morphology, beverage pH, and colorant composition as key considerations when selecting universal-shade composites for patients with frequent exposure to acidic or chromogenic beverages.

## Figures and Tables

**Table 1:** Table 1: Study materials and immersion media. Panel A: Resin-based composite materials evaluated. Panel B: Immersion media, with distilled water listed first as the standard ISO 4049:2019 storage medium.

**A. Resin-Based Composite Materials**				
**Composite**	**Manufacturer**	**Shade**	**Resin Matrix**	**Filler System**
Omnichroma	Tokuyama Dental, Tokyo, Japan	Universal	UDMA, TEGDMA	Uniform 260 nm supra-nanospherical silica-zirconia; 69 vol%
Transcend	Ultradent Products Inc., South Jordan, UT, USA	UB (Universal Body)	Dimethacrylate resin, methacrylate resin	Silica, silane-treated silica nanofillers; Resin Particle Match™ technology
**B. Immersion Media**				
**Medium**	**Flavor**	Approx. pH*	**Sugar / 12 oz**	**Key Constituents & ISO Role**
Distilled water	-	≈7.0	0 g	Standard storage medium per ISO 4049:2019; no chromogens; quantifies water sorption & hydrolysis alone
Gatorade	Lemon Lime	≈2.9-3.2	21 g	Citric acid; Yellow #5 (tartrazine), Yellow #6; NaCl, KCl; sucrose, dextrose
C4 Energy Drink	Blue Bombsicle	≈3.0-3.5	0 g (sugar-free)	Citric acid, malic acid; Red 40, Blue 1; caffeine 200 mg; citrulline, sucralose

UDMA: Urethane dimethacrylate; TEGDMA: Triethylene glycol dimethacrylate; UB: Universal Body shade. *pH values are approximate and were obtained from manufacturer product information rather than measured experimentally; fresh solutions were prepared at each measurement time point. Objective instrumental characterization of beverage color was not performed.

**Table 2:** Table 2: Estimated marginal means (EMM) ± SE and Tukey HSD groupings for overall color change (ΔE00) by composite, time point, and immersion medium.

**Factor**	**Level**	**EMM ± SE**
Composite	Omnichroma	1.603 ± 0.056^A^
	Transcend	1.942 ± 0.056^B^
Time Point	Baseline (T0)	0.896 ± 0.126^A^
	Day 1 (T1)	1.220 ± 0.072^A^
	Day 3 (T2)	1.995 ± 0.072^B^
	Day 7 (T3)	2.395 ± 0.072^C^
Immersion Medium	Water	1.021 ± 0.072^A^
	Gatorade	2.281 ± 0.072^B^
	C4 Energy Drink	2.309 ± 0.072^B^

Baseline (T0) reflects inherent within-group color variation rather than post-immersion color change and is reported separately from the post-immersion time points (Day 1-Day 7); see the text for baseline values. D65 illuminant, using the CIE 2° standard observer. Levels sharing the same Tukey letter within a factor do not differ significantly (α=.05). Composite comparison uses Bonferroni adjustment.

**Table 3:** Table 3: Mean ± SD for ΔL*, Δa*, and Δb* by composite and immersion medium, collapsed across all time points (n=36 per composite × medium combination).

	ΔL* (lightness)			Δa* (green-red)			Δb* (blue-yellow)		
**Composite**	**Water**	**Gator.**	**C4**	**Water**	**Gator.**	**C4**	**Water**	**Gator.**	**C4**
Omnichroma	+0.47^a^ (±0.92)	+1.17^a^ (±1.85)	+0.89^a^ (±1.61)	+0.06^a ^(±0.29)	-0.03^a ^(±0.36)	+0.03^a ^(±0.39)	+1.32^b^ (±0.44)	+2.33^a^ (±1.17)	-1.58^c ^(±1.57)
Transcend	-0.40^b^ (±1.25)	+1.52^a^ (±1.28)	-0.18^b^ (±2.41)	+0.01^a ^(±0.18)	-0.45^c ^(±0.71)	+0.45^a ^(±0.35)	+0.17^b^ (±0.63)	+2.48^a ^(±2.13)	-2.83^c ^(±2.31)

Within each axis block, columns give the three immersion media (Water, Gatorade, C4); the upper figure in each cell is the mean shift with its Tukey HSD subset letter and the lower figure is the standard deviation. Positive ΔL*: Lightening; positive Δa*: A shift toward red; positive Δb*: A shift toward yellow. Lowercase letters denote homogeneous subsets within each axis column (α=.05); values sharing a letter do not differ significantly. This table summarizes the overall shift on each axis and does not resolve change over time; the time course of each parameter is reported in the text.

**Table 4:** Table 4: Mean ΔE_00_ (±SD; n=12 per group) with Paravina et al. [10] clinical classifications for each group at each time point.

**Group**	**Composite**	**Medium**	**Day 1 (T1)**	**Day 3 (T2)**	**Day 7 (T3)**
OW	Omnichroma	Water	Acceptable 1.27 (±0.33)^A^	Acceptable 0.93 (±0.36)^A^	Acceptable 1.24 (±0.29)^A^
OG	Omnichroma	Gatorade	Moderate 1.81 (±0.69)^B^	Moderate 2.44 (±0.74)^B^	Moderate 2.29 (±0.31)^B^
OC	Omnichroma	C4 Energy	Acceptable 1.49 (±0.56)^A^	Moderate 1.83 (±0.36)^B^	Moderate 2.09 (±0.82)^B^
TW	Transcend	Water	Excellent 0.55 (±0.43)^A^	Acceptable 0.86 (±0.53)^A^	Acceptable 1.29 (±0.75)^A^
TG	Transcend	Gatorade	Excellent 0.61 (±0.29)^A^	Moderate 2.78 (±0.36)^C^	Clearly Unacceptable 3.76 (±0.50)^C^
TC	Transcend	C4 Energy	Acceptable 1.59 (±0.33)^A^	Moderate 3.14 (±0.91)^C^	Clearly Unacceptable 3.71 (±0.33)^C^

Values are mean ΔE_00_ (±SD; n=12 per group). The clinical grade printed above each value indicates the Paravina et al. [10] clinical threshold for that group: Excellent (ΔE_00_<0.8). Acceptable (0.8≤ΔE_00_<1.8). Moderate (1.8≤ΔE_00_≤3.6). Clearly Unacceptable (ΔE_00_>3.6). Uppercase letters denote homogeneous subsets from pairwise comparisons within each time point across all six groups, based on the pooled error term from the three-way ANOVA (MSE=0.378, df=220; α=.05). Groups sharing the same letter do not differ significantly; groups with different letters differ significantly (p<.05).

**Figure 1:**
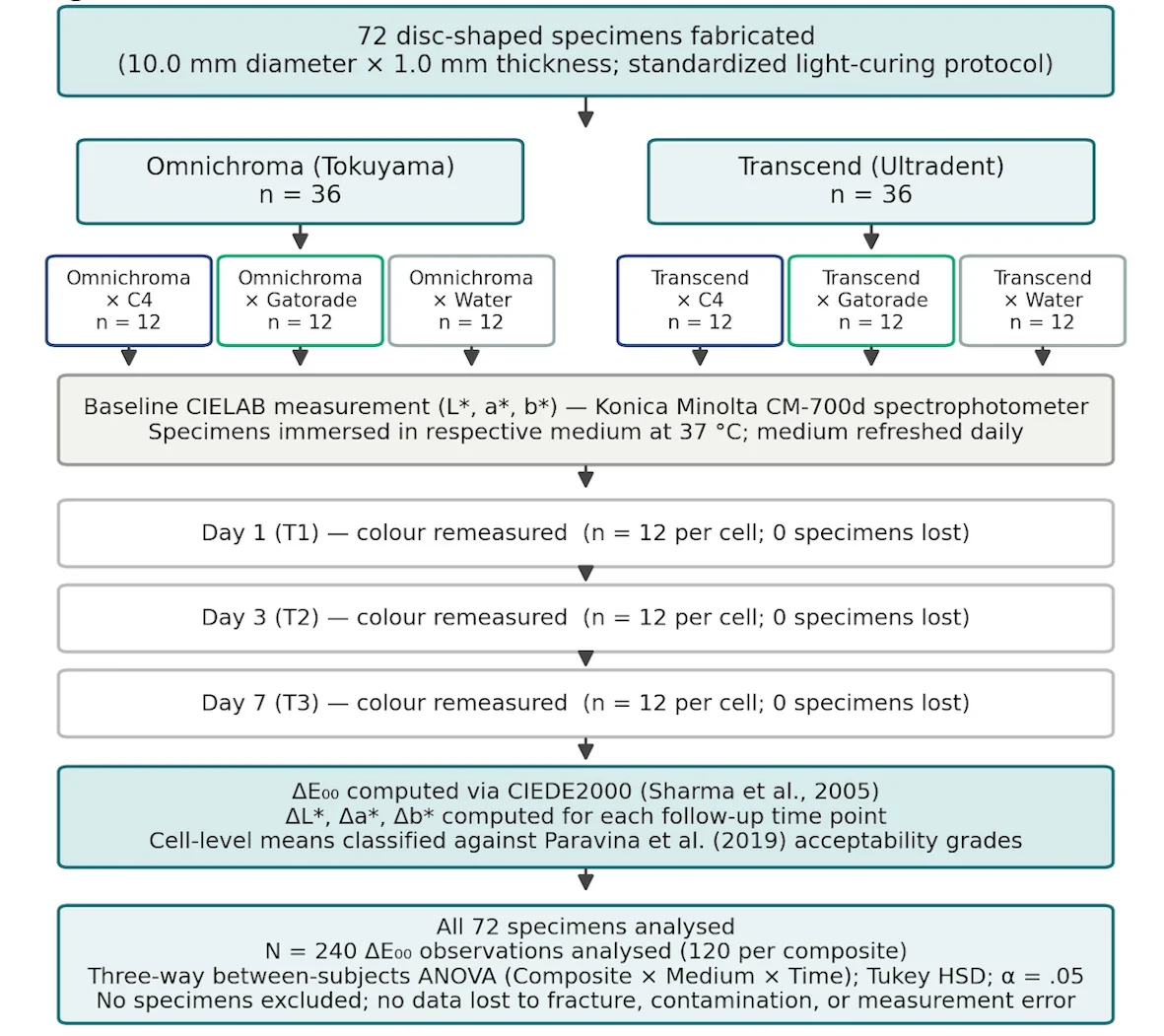
Fig. 1: CONSORT-style flow of all 72 specimens through enrollment, group allocation, baseline measurement, follow-up color measurement, and final analysis. No specimens were excluded and no data were lost.

**Figure 2:**
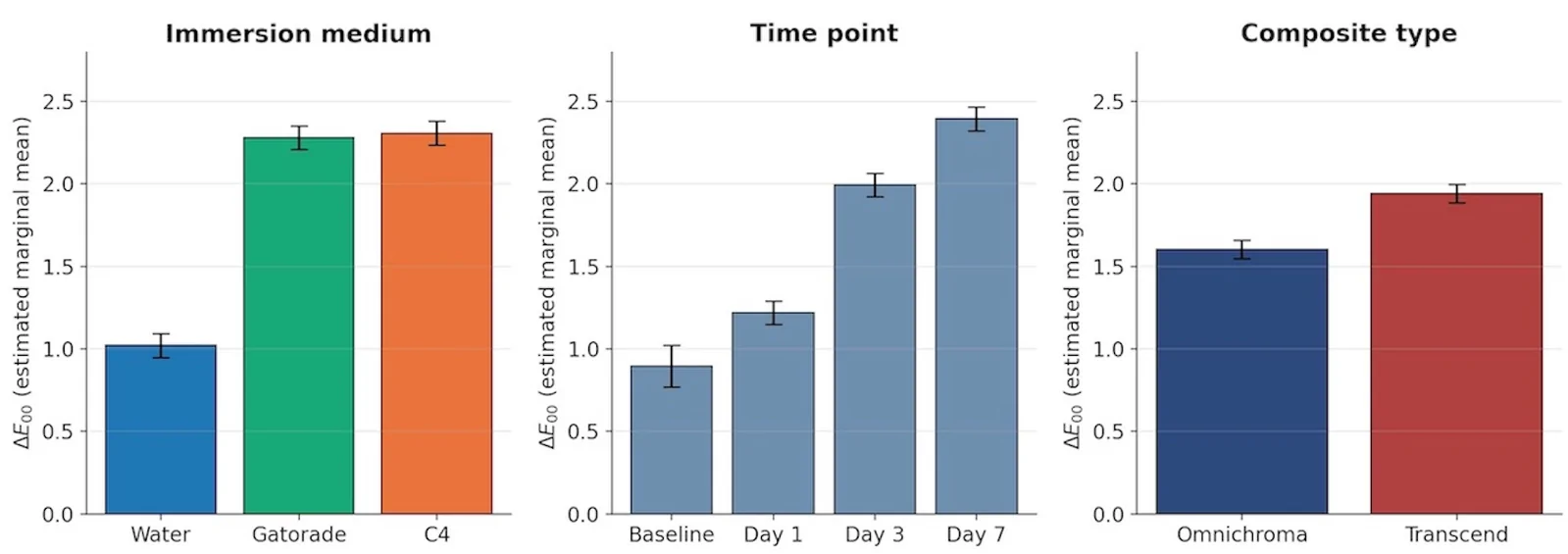
Fig. 2: Estimated marginal means of ΔE00 for the main effects of immersion medium, time point, and composite type. Error bars represent ±1 standard error.

**Figure 3:**
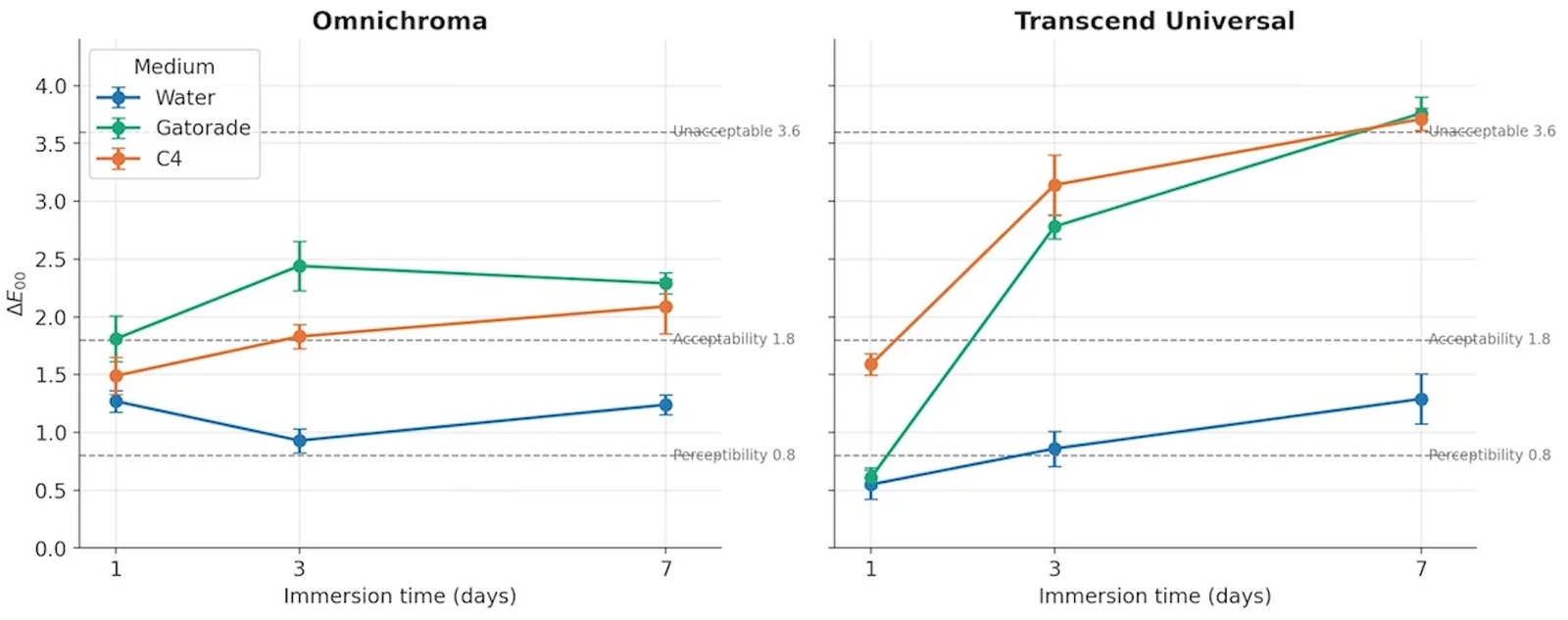
Fig. 3: Composite × Time interaction profiles for ΔE00 across the three immersion media (Omnichroma and Transcend panels). Dashed horizontal lines indicate the Paravina et al. [10] interpretation grades: The perceptibility (0.8) and acceptability (1.8) thresholds, with the 3.6 line marking the boundary between moderately and clearly unacceptable mismatch. Error bars represent ±1 standard error.

## Data Availability

The data used and/or analyzed during the current study are available from the corresponding author on reasonable request.
